# Understanding antimicrobial susceptibility profile of *Finegoldia magna*: an insight to an untrodden path

**DOI:** 10.1186/s12941-023-00583-1

**Published:** 2023-04-25

**Authors:** Seema Shetty, Renuka Anegundi, Padmaja Ananth Shenoy, Shashidhar Vishwanath

**Affiliations:** 1 Department of Microbiology, Kasturba Medical College, Manipal, Manipal Academy of Higher Education, Manipal, Karnataka 576104 India; 2 Manipal Centre for Infectious Diseases, Prasanna School of Public Health, Manipal Academy of Higher Education, Manipal, Karnataka, India

**Keywords:** Anaerobic cocci, *Finegoldia magna*, Antimicrobial resistance, Metronidazole, Clindamycin Resistance

## Abstract

**Background:**

*Finegoldia magna* (formerly known as *Peptococcus magnus* or *Peptostreptococcus magnus*) belonging to phylum Firmicutes, class Clostridia and genus Finegoldia, is the only species known to cause infections in human beings. Amongst Gram positive anaerobic cocci, *F. magna* is known to be the most virulent with a high pathogenic potential. Significant upsurge in antimicrobial resistance among anaerobes has been documented by various studies. *F. magna* is known to be susceptible to most of the anti-anaerobic antimicrobials, however, multidrug resistant strains are being reported in literature. The present study was undertaken to highlight the role of *F. magna* in clinical infections and to analyze their antimicrobial susceptibility patterns.

**Methods:**

The present study was conducted in a tertiary care teaching hospital in Southern India. 42 clinical isolates of *F. magna* recovered from diverse clinical infections between January 2011 to December 2015 were studied. These isolates were subjected to antimicrobial susceptibility testing against metronidazole, clindamycin, cefoxitin, penicillin, chloramphenicol and linezolid.

**Results:**

Among the 42 isolates studied, majority of them were revived from diabetic foot infections (31%) followed by necrotizing fasciitis (19%) and deep-seated abscesses (19%). All the *F. magna* isolates showed good in-vitro activity against metronidazole, cefoxitin, linezolid and chloramphenicol. Clindamycin and penicillin resistance were observed against 9.5% and 2.4% of the isolates respectively. However, β-lactamase activity was not detected.

**Conclusion:**

The antimicrobial resistance among anaerobes varies from pathogen to pathogen and region to region. Hence, a deep understanding of resistance pattern is necessary for better management of clinical infections.

## Introduction

Gram positive anaerobic cocci (GPAC) form an integral part of normal human microbiota colonizing surfaces of skin, mouth, gastrointestinal and urogenital system [1]. These GPAC are often considered as opportunistic pathogens and are significantly recovered from diverse clinical infections constituting 25–30% of all clinical anaerobic isolates [1–3]. The most commonly isolated GPAC in infectious clinical materials include *Peptostreptococcus anaerobius*, *Finegoldia magna*, *Peptoniphilus asaccharolyticus* and *Parvimonas micra*. *F. magna* is one of the most common anaerobic pathogens accounting to 5–12% of all anaerobic isolates and 20–38% of all GPAC [3].

The genus Finegoldia belongs to the phylum Firmicutes, class Clostridia and is named after the American Microbiologist S. M. Finegold [1, 2]. *F. magna* (formerly known as *Peptococcus magnus* or *Peptostreptococcus magnus*) is the lone species of clinical importance in this genus [2]. *F. magna* is known to cause a variety of clinical infections ranging from deep-seated infections to life-threatening conditions such as endocarditis, prosthetic joint infections and necrotizing pneumonia [1–4]. The infections associated with GPAC are usually polymicrobial in nature, however, *F. magna* is often isolated in pure cultures [4]. Several virulence factors produced by this pathogen are known to influence disease pathogenesis [1–3].

Antimicrobial resistance among anaerobes is witnessing a significant upsurge in the recent years worldwide [5]. Though, GPAC are usually susceptible to commonly used anaerobic antimicrobials, increasing resistance trends and significant difference in susceptibility profile among different species of GPAC have been reported in literature [5, 6]. Variable resistance to penicillin (7–10%), metronidazole (5–10%) and clindamycin (7–20%) have been reported amongst GPAC [1, 7]. *F. magna* has shown lower resistance rates (10–20%) to clindamycin, metronidazole and penicillin, while higher resistance rates (> 20%) have been demonstrated against erythromycin and tetracycline [2]. Owing to these study findings, it becomes very essential to diagnose and identify isolates from diverse clinical infectious materials up to species level.

Unlike aerobic counterparts, the anaerobic culture and susceptibility testing is not routinely performed in most of the clinical laboratories due to its stringent culture techniques, cost-effectiveness and lack of expertise [8]. As most of the anaerobic infections are polymicrobial in nature, there is practice of using empirical antimicrobials such as metronidazole and clindamycin in treating infections in clinical settings. In view of reduced susceptibility to penicillin, clindamycin and metronidazole among GPAC, the usage of empirical antimicrobial therapy may not be of clinical benefit but in turn would pose an increased burden on patient care and economic consequences. In addition, rampant over-the-counter usage of empirical antimicrobials with lack of awareness regarding their increasing resistance trends can result in rise and spread of multidrug resistant anaerobic superbugs [1, 2, 9]. In this regard, the knowledge and availability of antimicrobial resistance data becomes crucial to tackle the emergence and transmissibility of antimicrobial resistance. The present work was undertaken to highlight the role of *F. magna* in clinical infections with special reference to their antimicrobial resistance patterns.

## Materials and methods

### Study population and study design

The present study was conducted in the Department of Microbiology, a constituent of a tertiary care teaching hospital in Southern India. A total of 42 clinical isolates of *F. magna* which were recovered from diverse clinical infections between January 2011 to December 2015 were included in this study. All these strains were isolated from various clinical specimens including pus aspirates, soft tissue specimens, bone and body fluids, obtained from diverse infectious sites and were stored at -80◦C in skimmed milk broth until further analysis.

### Sample processing and identification

The stored isolates were revived on anaerobic blood agar (HiMedia Labs, Mumbai, India) and were incubated for 72 h at 37◦C in Whitley A35 Anaerobic workstation (Don Whitley Scientific, Shipley, UK). Prior to antimicrobial susceptibility testing, the identification of the isolates was confirmed by Matrix‑assisted laser desorption/ionization‑time of flight mass spectrometry (Vitek MS, bioMerieux Inc., France). β‑lactamase production was detected using nitrocephin impregnated paper disks (BD BBL Cefinase, Becton Dickinson and Co, Sparks, USA) [10].

### Antimicrobial susceptibility testing

The minimum inhibitory concentrations (MIC) of *F. magna* isolates were determined by reference agar dilution method and/or antimicrobial gradient diffusion method (E test, bioMerieux Inc., Marcy L’Etoile, France). The antimicrobial susceptibility was determined against six antimicrobial agents, of which metronidazole, clindamycin, cefoxitin, penicillin and chloramphenicol were tested using agar dilution method, while linezolid was analyzed using E test method. The agar dilution (Wadsworth method) was performed following Clinical and Laboratory Standard Institute (CLSI) guidelines M11-A6 (CLSI 2013) [11] on Wilkin-Chalgren agar with Gram-positive anaerobic supplement (HiMedia Labs, Mumbai, India). The inoculum for each isolate was prepared to adjust the turbidity to 1.0 McFarland standard. The inoculated plates were incubated in anaerobic atmosphere for 48 h. The lowest concentration at which a marked reduction in the growth was observed, was considered as the MIC of the individual antimicrobial agent. The E test was performed on anaerobic blood agar as per manufacturer’s guidelines and results were read after 48 h of incubation. MIC was recorded at the point where the elliptical zone intersected with the strip. The MIC value was interpreted as per the CLSI guidelines M11-A6 (CLSI 2013) [10]. *B. fragilis* ATCC 25285 was used as the reference strain for quality control of susceptibility testing.

## Results

Majority of *F. magna* isolates were recovered from diabetic foot infections (n = 13, 31%) followed by deep-seated abscesses (n = 8, 19%) and cases of necrotizing fasciitis (n = 8, 19%). The other clinical conditions noted were chronic osteomyelitis (n = 6), chronic non-healing ulcer (n = 3), wet gangrene (n = 3) and chronic suppurative otitis media (n = 2). Deep-seated abscesses included intra-abdominal abscess (n = 4), puerperal breast abscess (n = 1), abdominal wall abscess (n = 1), perianal abscess (n = 1) and hand abscess (n = 1). Of the 42 *F. magna* isolates, 21 showed monomicrobial anaerobic growth and were majorly isolated from cases of diabetic foot infections, deep-seated abscesses and chronic osteomyelitis.

Good in-vitro susceptibility was observed in all the isolates of *F. magna* against metronidazole, cefoxitin, linezolid and chloramphenicol. A marked resistance towards clindamycin was observed in 9.5% (n = 4) isolates while only one strain was found to be resistant to penicillin (2.4%). β-lactamase activity was not detected in any of the isolates. Table 1 illustrates the MIC50 and MIC90 values of tested antimicrobial agents and their susceptibility patterns in *F. magna*.

**Table 1:** MIC50 and MIC90 values of tested antimicrobial agents and their susceptibility patterns in *F. magna*

Antimicrobial agent	MIC (µg/mL)			S (%)	R (%)
	MIC_50_	MIC_90_	Range		
Penicillin	0.125	0.5	0.125-4	97.6	2.4
Cefoxitin	2	4	2–4	100	-
Metronidazole	0.5	4	0.25-8	100	-
Clindamycin	2	4	0.25-16	90.5	9.5
Chloramphenicol	1	2	0.25-4	100	-
Linezolid	0.25	0.5	0.016-0.5	100	-

MIC, minimum inhibitory concentration; MIC50/90, MIC values for 50% and 90% of the organisms; S, susceptible; R, resistant

## Discussion

*F. magna* is a clinically important GPAC with high pathogenic potential. It is frequently recovered from soft tissue infections, diabetic foot infections, deep-seated abscesses, bone and joint infections [1, 2, 4–6]. In this study, majority of the isolations were achieved from diabetic foot infections (31%), necrotizing fasciitis (19%) and deep-seated abscesses (19%). *F. magna* is known to elaborate range of putative virulence factors including protein L, peptostreptococcal albumin binding protein (PAB), subtilisin-like proteinase (SufA) and *F. magna* adhesion Factor (FAF) in addition to production of collagenase enzyme. The collagenase production leads to breakdown of collagen which is abundantly present in skin, tendons, cartilage and bones. This results in loss of tissue integrity and breakdown of amino acids there by producing favorable environment for growth and multiplication of asaccharolytic organism like *F. magna.*. There are reports mentioning the ability of *F. magna* in producing biofilms which may in turn interfere with the targeted antimicrobial therapy [1, 12, 13]. Thus, better understanding of the bacterial properties and their virulence mechanisms would assist the clinicians in accurate treatment and management of these infections.

Performing the antimicrobial susceptibility testing of anaerobic bacteria would be an expensive affair requiring experienced laboratory staff and adequate resources which may not be feasible in all settings. The agar dilution method, broth microdilution method or gradient tests (E test, spiral gradient test) are the various methods used for determining MICs for anaerobic organisms [8]. The practice of incorporating metronidazole disk (5 µg) in routine anaerobic culture plates and further testing of those isolates (showing zone size of less than 15 mm) for aerotolerance tests can rule out the presence of facultative anaerobic bacteria [3, 8]. The European Committee on Antimicrobial Susceptibility Testing (EUCAST) had proposed disk diffusion susceptibility testing for anaerobes, which was found to be beneficial in fast growing organisms like *B. fragilis*. However, in view of poor growth and varied results (in comparison to reference agar dilution method), this technique is yet to be standardized for testing GPAC [14].

Antimicrobial resistance trends among anaerobes is highly diverse and dynamic, variability being observed between species, regions and clinical set ups. Metronidazole, a 5-nitroimidazole derivative is a common and long known antimicrobial for empirical therapy. Several complex mechanisms are implicated in the development of metronidazole resistance. There are reports of increasing resistance trends towards metronidazole among *B. fragilis* group. In contrast, *F. magna* is known to be susceptible to the commonly used anti-anaerobic antimicrobials [1, 2]. However, some studies have highlighted the resistance to metronidazole among GPAC including *F. magna* [15, 16]. In this study, none of the isolates showed resistance to metronidazole which was in line with other studies [13, 17–20]. However, this study did not focus on analyzing the genetic mechanisms of drug resistance.

Clindamycin is another empirical drug commonly used in treatment of anaerobic infections [14]. Variable rates of clindamycin resistance have been mentioned in literature ranging between 3 and 51% [5, 6, 15, 18, 21, 22]. This high resistance rates towards clindamycin could be attributed to alteration in target site by RNA methylase and presence of erm gene [5]. Compared to other studies, we noted a decreased resistance rate (9.5%) towards clindamycin in our setup.

GPAC are generally known to be susceptible to β-lactam group, β-lactam β-lactamase inhibitors, carbapenems and cephalosporins. In the present study, one *F. magna* isolate was found to be resistant to penicillin (2.4%) although no β-lactamase activity was demonstrated. There are varied reports for in-vitro activity of β-lactams among anaerobic bacteria with high rates of resistance being depicted in *B. fragilis* group [23]. Although good in-vitro activity of penicillin has been noted in *F. magna*, the resistance rates towards β-lactam group has been reported among other GPAC, particularly in *P. anaerobius* isolates [5]. Chloramphenicol, although not used routinely [9], has shown good susceptibility rates among anaerobic genera with an exception to study by Lee et al. [18] where two isolates of *F. magna* (n = 15) showed high MIC values (16–32 mg/L) and were found to be resistant. Good linezolid activity has been depicted in literature against GPAC which was concordant with our study [5, 7, 17, 24]. Multidrug resistant *F. magna* have been emerging and are being reported in some studies. In a study conducted by Shilnikova and Dmitrieva, a multidrug resistant *F. magna* was reported from mediastinal tumor showing resistance to metronidazole, ciprofloxacin, levofloxacin, penicillin G and intermediate resistance to amoxicillin-clavulanate [7].

## Conclusion

*F. magna* was isolated from diverse clinical infection sites in our study. The isolates were found to be largely susceptible to various antimicrobial agents with anaerobic coverage except for clindamycin. Being a frequent pathogen in infections involving anaerobes and with reports of emerging resistance from across the globe including multi-drug reistance, it becomes essential to monitor the susceptibility trends of *F. magna*. Local knowledge of the resistance patterns of GPAC including *F. magna* and their inclusion in institutional antibiograms shall aid in better patient management.

## Data Availability

All data generated or analyzed during this study are included in this manuscript.

## Abbreviations

GPAC: Gram positive anaerobic cocci
MIC: minimum inhibitory concentration
CLSI: Clinical and Laboratory Standard Institute
PAB: peptostreptococcal albumin binding protein
SufA: subtilisin-like proteinase
FAF: *F. magna* adhesion Factor
EUCAST: European Committee on Antimicrobial Susceptibility Testing
